# Effect of a short training on neonatal face-mask ventilation performance in a low resource setting

**DOI:** 10.1371/journal.pone.0186731

**Published:** 2017-10-26

**Authors:** Alessandro Mazza, Francesco Cavallin, Anita Cappellari, Antuan Divisic, Ivana Grbin, Jean Akakpo, Abdou Razak Moukaila, Daniele Trevisanuto

**Affiliations:** 1 Department of Women’s and Children’s Health, University of Padua, Azienda Ospedaliera di Padova, Padova, Italy; 2 Independent Statistician, Padova, Italy; 3 Maternitè, Marie Mère de la Providence, Centre Médico-Social, Kouvè, Togo, Africa; University of Bari, ITALY

## Abstract

**Background:**

We assessed whether a short training, effective in a high resource country, was able to improve the quality of face-mask ventilation (FMV) in a low resource setting.

**Methods:**

Local healthcare providers at the Centre Médico-Social, Kouvè, Togo were asked to ventilate a neonatal leak-free manikin before (time—t_1_) and after (t_2_) a two-minute training session. Immediately after this section, a further two-minute training with participants aware of the data monitor was offered. Finally, a third 1-minute FMV round (t_3_) was performed by each participant. Ventilatory parameters were recorded using a computerized system. Primary outcome was the percentage of breaths with relevant mask leak (>25%). Secondary outcomes were percentages of breaths with a low peak inspiratory pressure (PIP<20 cm H_2_O), within the recommended PIP (20–35 cm H_2_O) and with a high PIP (>35 cm H_2_O).

**Results:**

Twenty-six subjects participated in the study. The percentage of relevant mask leak significantly decreased (p<0.0001; β = -0.76, SE = 0.10) from 89.7% (SD 21.5%) at t_1_ to 45.4% (SD 27.2%) at t_2_ and to 18.3% (SD 20.1%) at t_3_. The percentage of breaths within the recommended PIP significantly increased (p<0.0001; β = +0.54, SE = 0.12). The percentage of breaths with PIP>35 cm H2O was 19.5% (SD 32.8%) at t_1_ and 39.2% (SD 37.7%) at t_2_ (padj = 0.27; β = +0.61, SE = 0.36) and significantly decreased (padj = 0.01; β = -1.61, SE = 0.55) to 6.0% (SD 15.4%) at t_3_.

**Conclusions:**

A 2-minute training on FMV, effective in a high resource country, had a positive effect also in a low resource setting. FMV performance further improved after an extra 2-minute verbal recall plus real time feedback. Although the training was extended, it still does not cost much time and effort. Further studies are needed to establish if these basic skills are transferred in real patients and if they are maintained over time.

## Introduction

Every year around 6,6 million children worldwide under 5 year die. Of these, 44% are in the neonatal period. Intrapartum-related events (“birth asphyxia”), account for a quarter of neonatal deaths suggesting that basic skill training of those involved in the care of neonates at delivery is a crucial investment [1–3].

Recently, neonatal resuscitation is receiving increasing attention as a missed opportunity to improve morbidity and mortality outcomes. Newton and English reviewed the evidence for neonatal resuscitation and concluded that effective resuscitation in low-resource settings was possible with basic equipment and skills [4]. Training health care providers in neonatal resuscitation may prevent 30% of deaths of full-term babies experiencing adverse intrapartum events, as well as 5%–10% of deaths among infants born preterm [5,6].

Effective positive pressure ventilation (PPV) is the most important intervention for successful resuscitation of the newborn [7,8]. In the settings where continuous gas flow is not available, PPV is administered by using a self-inflating bag (SIB) and mask. However, achieving effective face-mask ventilation (FMV) can be difficult [9–12]. Specific key points of the procedure, such as reduce leak around the mask, avoid the airway block and administer adequate pressures, need to be well known by healthcare providers [7,8].

A previous study conducted in a high resource setting showed that a structured two-minute training based on 6 key-points significantly improved the quality of FMV in a manikin model. The authors suggested that this training could be incorporated into any educational program [13].

In addition, Kelm et al. demonstrated that a training scheme of FMV with a SIB, including a simple respiratory function monitor to feed back the level of the applied ventilator parameters to the individual operator significantly reduced the occurrence of excessive pulmonary pressures and volumes [14].

However, the impact of these training interventions in a low-resource setting remains unknown.

The aim of this study was to assess whether a short training, effective in high resource countries, was able to improve the quality of FMV in a low resource setting.

## Methods

### Setting

This study was conducted at the Centre Médico-Social, Kouvè, Préfecture de Yoto, Togo. This is a rural hospital where about 600 deliveries occur every year.

### Study design

Participants consisted of physicians, midwives and nurses. Most of them attended a neonatal theoretical and practical resuscitation course two years before. All participants were asked to administer FMV for a minute to a manikin. A neonatal manikin (Laerdal Resusci Baby, Laerdal, Stavanger, Norway) was modified for obtaining a leak free system with a 50 ml test lung as previously described [13]. A 240 ml-self inflating bag and a size 1 round mask (Laerdal, Stavanger, Norway) were used to administer PPV. The bag had a pressure release valve at 35 cmH_2_O without end expiratory pressure (PEEP) valve.

PPV parameters (Ventilatory rate–VR, Peak Inspiratory Pressure—PIP, Flow, Inspiratory Volume -Vti-, Expiratory Volume -Vte-, Leak) were measured using a training computerized system for neonatal mask ventilation (NewLifebox-T—Neonatal Resuscitation Trainer, Advanced Life Diagnostics UG, Weener, Germany). It measures pressure and air flow through the resuscitation mask using a flow/pressure probe which is placed between the face-mask and the resuscitation device (dead space of 0,7 ml). Data were analyzed by a software installed on a standard computer (NewLifebox Training Center software, Advanced Life Diagnostics UG, Weener, Germany). All signals were digitized and recorded with a data acquisition program in the software. After installing the software, the Newlifebox-T device was connected to the computer using the USB-cable. The flow sensor was re-calibrated before starting each measurement. In this way, the sensors do not drift away from zero. The software indicates it. For this baseline test, the flow probe was connected to the NewLifebox-T, while absolutely no air flowed to the probe and no pressure was applied to the probe.

The air flow was integrated to provide inspired—and expired tidal volumes (Vti and Vte). Leak at the face-mask was calculated as the difference between the inspired and expired tidal volumes, expressed as a percentage of the inspired tidal volume (leak percentage = [(inspiratory tidal volume–expiratory tidal volume) / inspiratory tidal volume] x 100) [13].

The verbal instruction and the demonstration were decided and standardized before starting the study.

**Fig 1:**
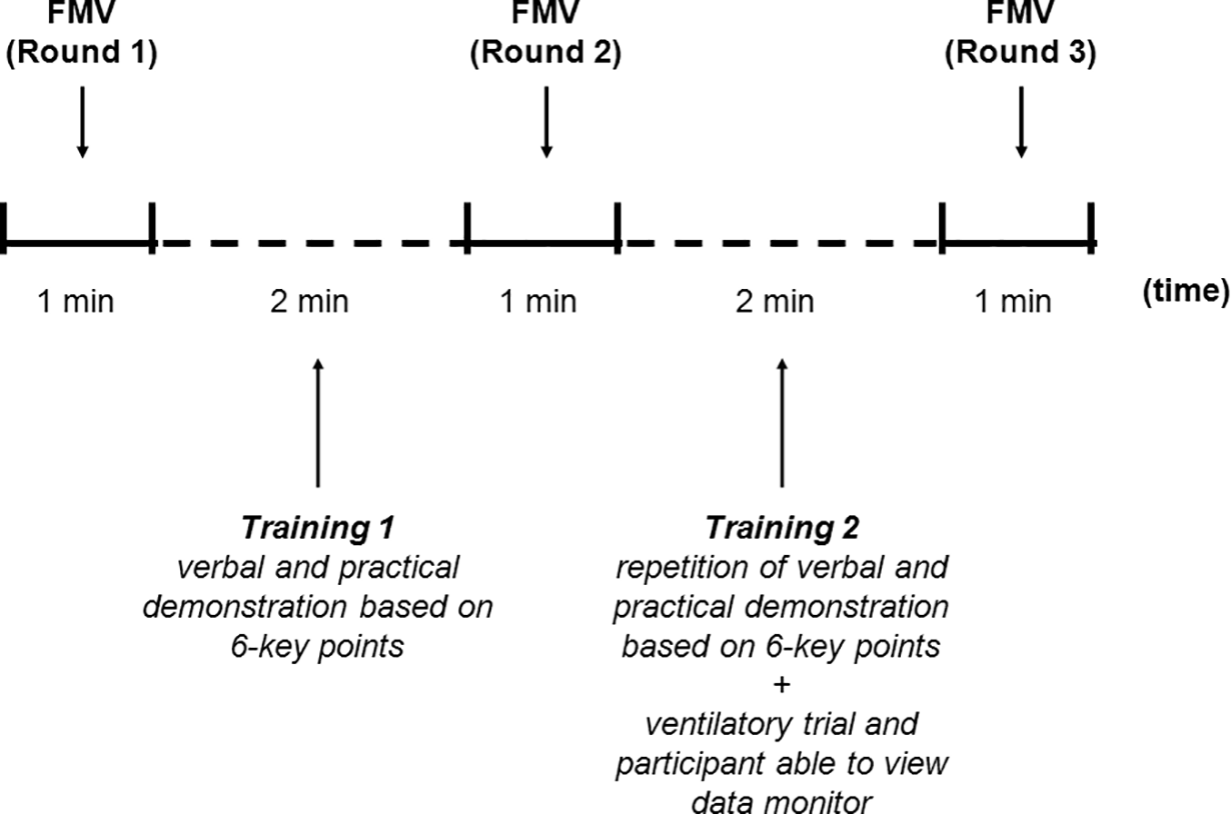
Study design. FMV—face-mask ventilation.

The experiment was subdivided into 5 steps, according to the following protocol (Fig 1):

1. At the beginning, participants ventilated the manikin without any instruction for 1 minute. Mask leak, PIP and VR were recorded during performance, but participants were unaware of these data. Each participant was allowed to ventilate once.
2. After this first round, participants received a verbal instruction and demonstration by one instructor. In agreement with a previous study; the training was based on 6 key points and the duration was approximately 2 minutes [10].
3. Immediately after the instruction, participants were asked to repeat the procedure for 1 minute once. Also during this round, participants were unaware of the ventilatory parameters recorded by the software.
4. At this point, a second two-minute training, including the verbal recall of the 6-points scheme and a short ventilatory trial with participant aware of the data monitor, was offered to each participant.
5. Immediately after this second training, a third FMV round lasting 1 minute was performed by each participant, who remained blind to the data monitor.

#### Key points of the face-mask ventilation

All participants received two verbal instructions by one instructor. The following key points were explained and recommended:

1. positioning of the head in sniffing position;
2. positioning mask on the tip of chin, mouth and the nose, but not on the eyes;
3. appling mask to face using mild downward pressure and lifting the mandible up toward the mask;
4. gentle squeeze of the bag aimed to obtain effective chest movements that correspond to a peak inspiratory pressures between 20–35 cmH20;
5. beware that when the pop off valve of the SIB releases (at 35 cm H2O) uncontrolled pressures can be given;
6. mask ventilation at a rate of 40–60 inflations per minute.

### Outcomes

Primary outcome was the percentage of breaths per minute with relevant mask leak (higher than 25%). Secondary outcomes were the number of inflations per minute (VR), the percentage of breaths per minute with a low PIP (below 20 cm H_2_O), the percentage of breaths per minute within the recommended PIP (between 20 and 35 cm H_2_O) and the percentage of breaths per minute with a high PIP (higher than 35 cm H_2_O).

### Ethics statement

The study was approved by the institutional review board (IRB) of the Centre Médico-Social, Kouvè, Togo. All staff members gave verbal consent to participate in the study; it was documented in an excel sheet. Consent procedure was approved by the local IRB.

### Statistics

Continuous data were expressed as mean and standard deviation (SD) or median and interquartile range (IQR). VR (number of breaths per minute) was expressed as count number, whereas the number of breaths per minute with relevant mask leak, the number of breaths per minute with a low peak inspiratory pressure, the number of breaths per minute with a recommended peak inspiratory pressure and the number of breaths per minute with a high peak inspiratory pressure were expressed as percentages on total number of breaths per minute.

These variables were recorded at 3 time points (before the training t_1_, after the training t_2_ and after the recall training t_3_) for each subject, thus they were evaluated using a Poisson regression model for repeated measurements. The models for the primary and secondary outcomes included also the logarithm of ventilatory rate as offset. The effect of possible confounders (previous participation to a course on neonatal resuscitation and age) were assessed by including such effects in the models. Bonferroni’s adjustment for multiple comparisons was used when appropriate.

Statistical analysis will be performed using R 3.2.2 software (R Foundation for Statistical Computing, Vienna, Austria) [15].

A p-value less than 0.05 was considered statistically significant.

## Results

### Participants

Twenty-six healthcare providers attended the training and were included in the study. There were 14 males and 12 females with a median age of 36 years (IQR 29–41). Five participants were physicians, 6 midwives and 15 nurses. Most of them (21 out of 26, 80.8%) had already attended a course on neonatal resuscitation in the previous 2 years.

### Primary outcome

**Fig 2:**
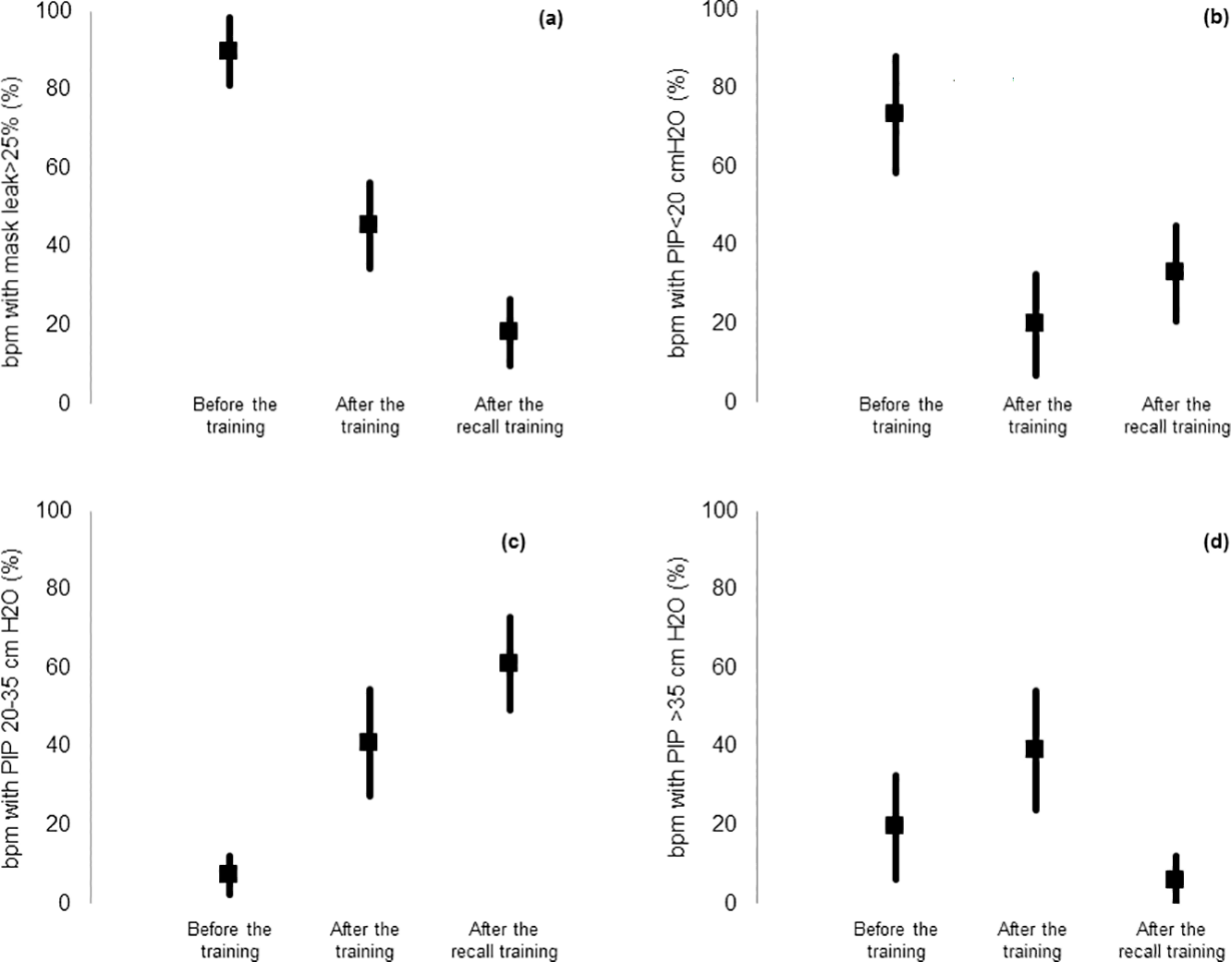
Percentage of breaths per minute (bpm) with (a) relevant mask leak (>25%), (b) low peak inspiratory pressure (PIP<20 cm H2O), (c) peak inspiratory pressure in the recommended range (PIP = 20–35 cm H2O), and (d) high peak inspiratory pressure (PIP>35 cm H2O). *Data are expressed as mean (95%CI)*.

Mean percentage of breaths per minute with relevant mask leaks (Fig 2A) significantly decreased (p<0.0001; β = -0.76, SE = 0.10) from 89.7% (SD 21.5%) at t_1_ to 45.4% (SD 27.2%) at t_2_ and to 18.3% (SD 20.1%) at t_3_. The effects of a previous course (p = 0.15), and age (p = 0.80) were not statistically significant.

### Secondary outcomes

Mean VR significantly decreased (p = 0.02; β = -0.17, SE = 0.05) from 58.7/min (SD 21.5) at t_1_ to 49.9/min (SD 14.7) at t_2_ and to 51.1/min (SD 11.4) at t_3,_ with a reduced effect if the participants had already attended a course (p = 0.08; β = 0.12, SE = 0.07). The effect of age (p = 0.82) was not statistically significant. Mean percentage of breaths per minute with a low PIP (Fig 2B) significantly decreased (p_adj_<0.0001; β = -1.38, SE = 0.31) from 73.3% (SD 36.5%) at t_1_ to 19.8% (SD 31.8%) at t_2_ then leveled (p_adj_ = 0.18; β = +0.60, SE = 0.32) to 32.9% (SD 30.4%) at t_3_. The effect of a previous neonatal course (p = 0.53) and age (p = 0.22) were not statistically significant.

Mean percentage of breaths per minute within the recommended PIP range (Fig 2C) significantly increased (p<0.0001; β = +0.54, SE = 0.12) from 7.1% (SD 12.3%) at t_1_ to 41.0% (SD 33.7%) at t_2_ and to 61.1% (SD 29.3%) at t_3_. This increment was enhanced if the participant had already attended a previous neonatal course (p = 0.04, β = +0.34, SE = 0.17), whereas the effect of age (p = 0.10) was not statistically significant.

Mean percentage of breaths per minute with a high PIP (Fig 2D) was 19.5% (SD 32.8%) at t_1_ and 39.2% (SD 37.7%) at t_2_ (p_adj_ = 0.27; β = +0.61, SE = 0.36) and significantly decreased (p_adj_ = 0.01; β = -1.61, SE = 0.55) to 6.0% (SD 15.4%) at t_3_. The effects of a previous neonatal course (p = 0.63) and age (p = 0.72) were not statistically significant.

## Discussion

Worldwide an estimated 3 to 6% of newborn infants need assisted PPV at birth [8]. It has been hypothesized that basic neonatal training programs improve neonatal survival [11,12]. As effective FMV is the most important intervention during neonatal resuscitation, all healthcare providers involved in the delivery room management of neonates have to be capable to perform this procedure [7,8]. Repetition of ventilation skills by a simple and short training may contribute to improve the basic neonatal resuscitation [5].

In this study, we assessed the efficacy of a short training program on the quality of FMV in a low resource setting. We found that the quality of manual ventilation, defined as mask leak and adequate PIP, significantly improved after the training.

A previous study, conducted in a high resource setting, showed that the quality of FMV in a manikin model improved significantly by using a structured 2-minute training consisting of 6 key-points [13]. In the first part of this study by using the same training program [13], we found a significant improvement on FMV performance, but the effectiveness of the performance was amplified when participants was offered a respiratory function monitor to feed back the level of the applied ventilatory parameters and the mask leak.

In comparison to the manikin study of Vonderen et al. [13], we modified the training a bit by adding an extra 2 minutes with verbal recall plus with monitor visible. This decision to use a respiratory function monitor was based on the positive results of a previous bench study conducted in a high resource setting [14]. These findings show that it only takes a few minutes to teach caregivers adequate mask ventilation, when using a monitor, and thus should be incorporated in every training.

Although the mask leak and the percentage of ventilatory breaths in the recommended PIP (between 20 and 35 cmH2O) improved after the initial training, this change was limited. In contrast with van Vonderen et al’s study [13], we noted that the percentage of ventilatory breaths in the “dangerous zone” (PIP>35cmH2O) significantly increased after the initial training. As an “aggressive” ventilation can give air-leak and pneumothorax that can be fatal in a setting where mechanical ventilation is not available, it is important to underline this risk following the training. In other words, the pre-training performance indicated the risk of hypoventilation of the manikin, but immediately after the first training, there was a risk of barotrauma. Both these situations should be avoided.

The quality of FMV significantly improved after the second section of 2-minute training, including the verbal recall of 6-key points and a short ventilatory trial allowing the participant to have a real time feedback of their performance. These results show that, in addition to the short training program suggested by previous work [13], an additional intervention was needed in a low resource setting to reach a good performance.

There are several reasons that need to be considered to explain the different impact of the same training on two groups of healthcare providers, including participants’ experience and setting. As the study was performed in a rural hospital with a low number of deliveries per year (about 600), it has to be recognized that caregivers had a low exposure to the procedure. However, we believe that our results can be translated to the majority of low-resource delivery centers where there is a limited number of births/year.

Our results cannot demonstrate whether the improvement registered after the second educational intervention was due to the verbal recall of the 6-key points or to the short ventilatory trial in which the participant was aware of the ventilatory parameters.

A further finding of this study was that participants who previously attended a neonatal theoretical and practical resuscitation course showed a better performance in comparison with those who did not suggesting that basic neonatal resuscitation skill may be maintined over time through repeated training [5,12].

The strength of this study is that it assessed the effect of a structured short training on the quality of FMV in a low resource setting. Nevertheless, it has some limitations that should be considered when interpreting the results. We enrolled a limited number of participants, but they represent the entire staff involved in the care of the newborns born in a typical organizational and cultural environment of a rural African delivery setting; our results could be different in other contexts. We evaluated the short-term effect of the intervention in a manikin model; it would be relevant to investigate the consequences of this training on the management of real patients. As previous work showed that low dose high frequency training programs reach the best results [12], it remains to be demonstrated if repetition of our intervention can further improve healthcare providers’ performance.

## Conclusions

A 2 minute structured training on FMV effective in a high resource country improved FMV performance in a low resource setting, but adding an extra session of 2 minutes with monitor visible led to further improvement. Although the training was extended, it still does not cost much time and effort. Further studies are needed to demonstrate whether participants’ skills are maintained over the time and whether our training model can improve FMV in clinical practice in a low-resource setting.

## Supporting information

S1 Table Supplementary data. (PDF)

## Abbreviations

FMV: face-mask ventilation
PIP: peak inspiratory pressure
PPV: positive pressure ventilation
SIB: self-inflating bag
VR: Ventilatory rate
