# Supplementary material for: Effect of a short training on neonatal face-mask ventilation performance in a low resource setting
Source: PLoS One. 2017 Oct 26;12(10):e0186731. doi: 10.1371/journal.pone.0186731 (PMC5658077; doi:10.1371/journal.pone.0186731)
Supplement: S1 Table — (PDF) [file pone.0186731.s001.pdf]

Id	Age	Sex	Job	Previous neonatal course	Breaths per minute 1	Breaths per minute 2	Breaths per minute 3	Relevant leaks 1	Relevant leaks 2	Relevant leaks 3	Low pip 1	Low pip 2	Low pip 3	Pip in range 1	Pip in range 2	Pip in range 3	High pip 1	High pip 2	High pip 3
1	39	m	nurse	yes	44	60	56	40	30	38	11	7	30	11	45	26	22	8	0
2	26	m	nurse	no	30	42	41	29	33	2	29	0	21	0	6	16	1	36	4
3	35	m	nurse	yes	40	38	44	37	30	16	18	0	6	3	2	38	19	36	0
4	50	f	nurse	yes	41	54	65	41	8	31	40	0	3	1	17	58	0	37	4
5	33	f	midwife	yes	65	93	65	55	88	NA	45	13	43	0	80	22	20	0	0
6	27	f	physician	yes	76	72	79	74	1	0	1	0	0	1	29	27	74	43	52
7	32	m	physician	no	73	45	53	71	35	37	0	5	43	5	11	10	68	29	0
8	28	f	midwife	yes	32	40	58	7	29	3	0	15	57	1	11	1	31	14	0
9	26	f	midwife	no	98	65	60	97	41	2	90	7	7	8	58	26	0	0	27
10	25	m	physician	yes	28	29	47	25	23	NA	28	0	5	0	0	37	0	29	5
11	38	m	nurse	yes	45	38	46	34	10	0	24	12	0	21	26	46	0	0	0
12	52	m	nurse	yes	30	52	53	30	3	3	30	0	3	0	10	42	0	42	8
13	26	m	nurse	yes	59	37	48	59	9	10	56	0	26	3	20	22	0	17	0
14	44	f	nurse	yes	33	31	27	33	16	9	33	31	9	0	0	18	0	0	0
15	50	f	nurse	no	43	31	32	43	28	6	42	31	5	1	0	26	0	0	1
16	51	m	nurse	yes	96	42	42	96	21	5	80	6	8	1	19	34	15	17	0
17	31	m	nurse	yes	70	49	45	70	12	0	70	0	0	0	13	45	0	36	0
18	43	f	nurse	yes	70	35	54	70	7	0	62	0	39	3	35	15	5	0	0
19	30	f	nurse	yes	52	60	35	52	30	4	52	2	23	0	41	12	0	17	0
20	37	f	midwife	yes	66	65	60	66	33	15	59	65	39	0	0	21	7	0	0
21	38	f	midwife	yes	84	46	60	84	9	7	84	19	5	0	27	55	0	0	0
22	32	m	nurse	yes	68	51	42	68	13	5	68	11	17	0	36	25	0	4	0
23	39	m	nurse	no	92	59	59	18	12	7	3	3	2	38	54	57	51	2	0
24	37	f	midwife	yes	80	46	45	60	12	5	76	1	5	4	32	40	0	13	0
25	29	m	physician	yes	64	64	54	61	31	14	53	6	36	11	1	18	0	57	0
26	41	m	physician	yes	46	54	59	45	22	0	42	0	2	4	0	57	0	54	0
